# Thrombocytopenia in severe iron deficiency anemia in children

**DOI:** 10.1002/hsr2.351

**Published:** 2021-09-17

**Authors:** Machiel van den Akker, Laura Chielens, Lisa Lopes, Jaques van Heerden, Mahmoud Zaqout, Jutte van der Werf Ten Bosch

**Affiliations:** ^1^ Department of Pediatrics ZNA Queen Paola Children's Hospital Antwerp Belgium; ^2^ Pediatric Hematology/Oncology Unit, Queen Mathilde Mother and Child Center Antwerp University Hospital Edegem Belgium; ^3^ Department of Pediatric Hematology Oncology UZ Brussel Jette Belgium; ^4^ Faculty of Medicine and Health Sciences University of Brussels Jette Belgium; ^5^ Faculty of Medicine and Health Sciences University of Antwerp Antwerp Belgium; ^6^ Pediatric Cardiology Unit, Queen Mathilde Mother and Child Center Antwerp University Hospital Edegem Belgium

**Keywords:** children, erythropoietin, iron deficiency anemia, thrombocytopenia, thrombocytosis

## Abstract

**Aim:**

Iron deficiency anemia (IDA) is common in the pediatric population and often accompanied by mild thrombocytosis, but rarely profound thrombocytopenia is seen. We describe the data of children with IDA and thrombocytopenia in two centers and discuss the published data in the literature.

**Methods:**

In this retrospective case series, the medical records of patients under the age of 19 years old diagnosed with IDA in two tertiary medical centers over the last 10 years, were reviewed. The data were collected and compared to the data published in the medical literature.

**Results:**

All the patients presented with severe IDA and thrombocytopenia improved with iron treatment. Although none of the patients had signs of major bleeding, the thrombocytopenia could mostly be classified as severe (platelet count <50×10E9/L). Due to the severity of the anemia, in about half of the cases, a red blood cell transfusion was given. The peak of the platelet count was seen in the first month after the start of iron treatment. In eight cases of children with IDA, the thrombocytopenia appeared after the supplementation of iron was started.

**Conclusion:**

Clinically stable children with severe IDA and thrombocytopenia, where other causes are very unlikely, warrant an empiric monotherapy with iron to prevent unnecessary investigations and treatments.

## 1 INTRODUCTION

Iron deficiency anemia (IDA) is often observed in the pediatric population, especially in children 1‐2 years old and adolescent girls.^1^ While in the younger age group, low dietary iron intake due to excessive whole milk consumption is often responsible for microcytic anemia, in the older group, besides low dietary iron, blood loss (like menorrhagia) can also be the cause of the IDA. We often see a mild thrombocytosis associated with IDA,^2^ which normalizes after iron substitution. A presentation of severe iron deficiency, a mild to severe thrombocytopenia can occasionally be observed,^3^ which in general resolved quickly after the start of iron therapy. Rarely the thrombocytopenia presents a few days after the start of iron replacement therapy. While studies involving adults with IDA and thrombocytopenia have been reported,^4^, ^5^, ^6^ data involving children is very limited. We present some unusual cases, provide an overview of related cases in the literature, and discuss the possible underlying etiology.

## 2 MATERIALS AND METHODS

In this retrospective case series, the medical records of patients under the age of 19 years diagnosed with IDA (microcytic anemia with hemoglobin ≦10 g/dL) and thrombocytopenia (platelets ≦100×10E9/L) in two tertiary medical centers (ZNA Paola Children's Hospital and University Hospital Antwerp, both in Antwerp, Belgium) over the last 10 years, were reviewed to gain more information about this rarely occurring entity. Patients with pre‐existing thrombocytopenia or with a disease explaining the thrombocytopenia at the time of diagnosis were excluded from the study. To compare our data to those from the literature, a PubMed search was conducted in the English‐language literature using the keywords “iron deficiency anemia” and “thrombocytopenia” in the age group 0‐18 years. The papers were reviewed and summarized in Table 1.

**TABLE 1:** Reported cases in the medical literature of IDA (Hb <7 g/dL) and thrombocytopenia (platelet count ≤100×10E9/L) in children (<19 y/o)

Author	Year	Age	Gender	Cause iron deficiency	Palpable spleen	Hb (g/dL)		MCV	Plt (×10E9/L)		WBC	Ferritin	Bone marrow (all no iron)			Therapy
						day 0	response (days)	(fL)	day 0	maximum (d)	(initial)	(ng/mL)	day	erythroid	megakaryocyte	Blood products (days), iron (days)
Lopas et al	1966	11 mo	M	Nutritional deficiency	2 cm	1.7	9 (D20)^×^	53	38	525 (D11)^×^	9.8	NR	D0	↓	↓	IM/PO
		2 y	F	Nutritional deficiency	no	2.1	10 (D16)^×^	52	42	750 (D12)^×^	10	NR	D0	↓	↓	IM/PO
		2 y	M	Nutritional deficiency	no	4	8.5 (D8)^×^	54	50	420 (D9)^×^	3	NR	D3	↑	↑	IM/PO
		3 y	M	Nutritional deficiency	2 cm	3.2	9 (D4)^×^	65	100	550 (D4)^×^	38	NR	D0	↑	n	RBC, PO
Soff et al	1988	17 y	F	Menorrhagia	minimal	3.2	NR	NR	168 (D6 21)	683 (D18)	2.7	<10	D0	n	n	RBC/Plt (D7), PO
Bruggers et al	1990	14 y	F	GI loss (HHT)	no	2.9	12.8 (D42)	65	65	normal (D5)	7.9	14.4	D0	↑	NR	RBC, PO
Perlman et al	2002	14 mo	M	Nutritional deficiency	no	2.2	NR	55	11	427 (D11)	5.3	?	D0	n	↑	PO
		15 mo	F	Nutritional deficiency	no	2.1	NR	61	35	174 (D2)	18.7	7	D0	↑	↑	RBC, PO
		25 mo	M	Nutritional deficiency	no	4.7	NR	49	64	695 (D8)	9.6	2	NR			PO
		30 mo	F	Nutritional deficiency	no	1.6	NR	60	89	125 (D1)	12.9	NR	NR			RBC, PO
		14 y	F	Menorrhagia	no	4.7	NR	59	64 (D3 ‐ 26)	125 (D7)	3.5	5	D0	↑	↑	RBC, PO
		17 y	F	Menorrhagia	no	2.9	NR	61	51	480 (D14	7.1	6	NR			RBC, PO
Morris et al	2010	18 mo	M	Nutritional deficiency	no	5.4	7.1 (D13)	53	18	1538 (D13)	14.2	17.7	NR			PO
		19 mo	M	Nutritional deficiency	no	2.3	10.3 (D24)	53	48	446 (D24)	7.5	2.9	NR			RBC, PO
		16 y	F	Menorrhagia	no	5.8	8.9 (D10)	60	7	802 (D10)	6	NR	NR			RBC/Plt(D1), PO
		18 y	F	Unknown	no	4.8	7.7 (D10)	57	46	1497 (D10)	4.7	2	D0	NR	NR	RBC, PO
Ozdemir et al	2011	2 y	M	NR	NR	5.3	NR	55	769 (D6 31)	382 (D12)	8.4	1.8	D0	NR	n	PO
		7 y	M	NR	no	6.9	7.4 (D5)	66	28	430 (D5)	2.9	10	D6	NR	n	PO
Jhamb et al	2011	16 y	F	Menorrhagia	NR	5	7.3 (D30)	58	60 (D3 ‐ 48)	1740 (D30)	2.6	NR	D3	n	n	PO
Alwazeer et al	2012	15 mo	M	Nutritional deficiency	NR	3.9	7.6 (D18)	52	177 (D7 ‐ 50)	954 (D18)	8.4	4	NR			RBC, PO
Otome et al	2014	16 y	F	Menorrhagia	no	5	9.5 (D11)	64	71 (D3 ‐ 27)	696 (D11)	3.7	2.4	D0	↑	↑	RBC, PO
Cunha et al	2015	16 y	F	Menorrhagia	no	5.8	8.1 (D9)	63	424 (D9 ‐ 45)	666 (D21)	5.4	3	D5	n	NR	RBC, IV(D1‐9)/PO
Giordano et al	2019	16 y	M	GI loss (ulceration)	NR	5.2	8.4 (D9)	54	40	277 (D10)	3.3	3.1	NR			RBC, PO
Huscenot et al	2019	17 y	F	Menorrhagia	NR	4.4	NR	59	33	1000 (D7)	NR	<3	D0	n	↑	RBC, IV
		16 y	F	Menorrhagia	NR	4.2	NR	65	21	321 (D7)	NR	2	NR			RBC, IV
Current paper	case 1	10 y	F	Nutritional deficiency	no	4.6	10.1 (D3)	66	38	719 (D9)	5	3	D0	↑	↑	RBC(D1), PO
	case 2	8 y	F	Nutritional deficiency	no	5.1	7.5 (D36)	56	18	801 (D9)	6.4	4	D1	↑	↑	PO
	case 3	15 y	M	Suspected GI loss	1 cm	4.1	9.3 (D28)	55	273 (D21 ‐ 34)	652 (D6)	5.8	<1	D21	↑	↑	RBC(D22)/Plt(D22), PO/IV(D23)
	case 4	14 y	F	Menorrhagia	no	6.7	13.2 (D42)	62	87	259 (D28)	4.5	2	NR			RBC(D14), PO
	case 5	17 y	F	Nutr. def. and menorrhagia	no	4.9	11.4 (D22)	62	16	741 (D13)	6.1	7	D1	n	↑	RBC(D1)/Plt(D1), PO/IV(D2)

*Note*: Cases of thrombocytopenia reported after the start of iron therapy are highlighted.

Abbreviations: D, day; F, female; GI, gastro‐intestinal; Hb, hemoglobin; HHT, Hereditary hemorrhagic teleangiectasia; IM, intramuscular; M, male; mo, month; MCV, mean corpuscular volume; NR, not reported and/or not done; Plt, platelets; PO, per os; RBC, red blood cell; WBC, white cell cell count; y, year; ^×^, according to graph.

### 2.1 Case presentations

**FIGURE 1:**
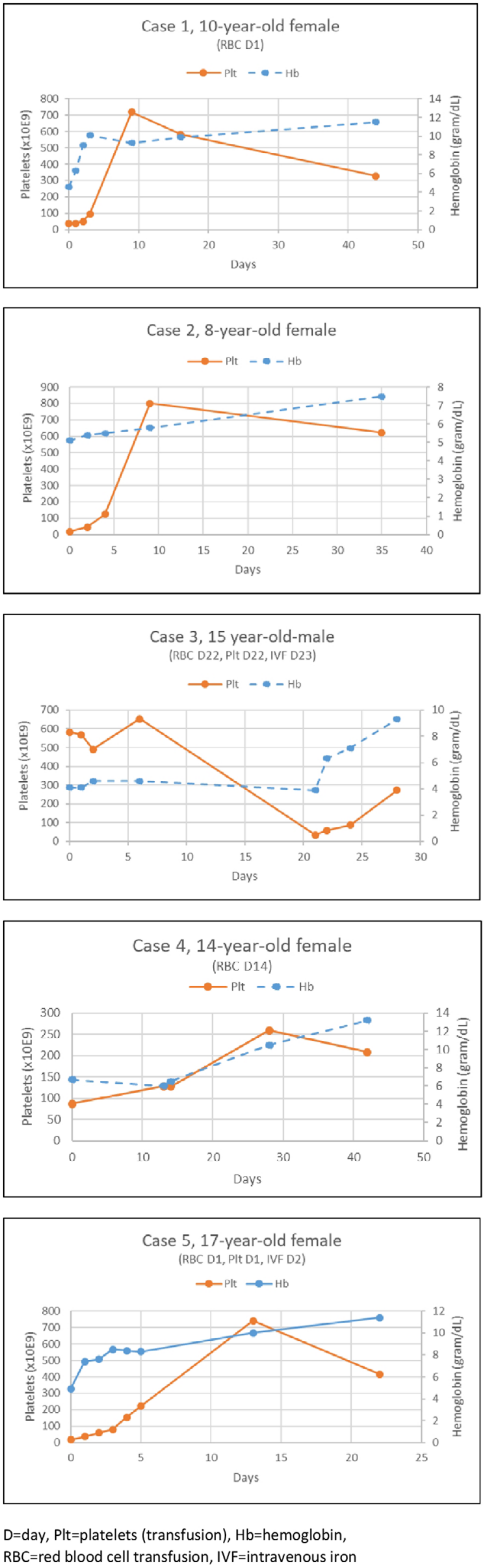
Five cases of iron deficiency anemia and thrombocytopenia reported in this article

Five patients were diagnosed during the study period. The clinical and laboratory characteristics are summarized here and visualized in Figure 1.

#### 2.1.1 Patient 1

A 10‐year‐old female was seen by the family physician for paleness, dizziness, and fatigue. Green vegetables and red meat were missing from her diet, while there was no menarche or history of suspected gastrointestinal blood loss. She was the second child of healthy, non‐consanguineous Moroccan parents. The family history was unremarkable. Physical examination revealed blood pressure 105/60 mmHg, pulse 105 per minute, respiratory rate 20 per minute, paleness, systolic murmur, and otherwise normal findings. The blood test showed severe microcytic anemia (Hb 4.6 g/dL, MCV 66 fL), severe thrombocytopenia (platelets 38×10E9/L), low ferritin (3 ng/mL), normal coagulations tests, negative parvovirus serology, and normal hemoglobin electrophoresis. Because two cell lines were down, a bone marrow investigation was performed, revealing the absence of iron staining and an increased megakaryocyte count. She received a red blood cell transfusion and oral iron medication was started (5 mg/kg/daily). Her blood cell counts improved rapidly.

#### 2.1.2 Patient 2

An 8‐year‐old female presented to the clinic because of paleness without complaints of fatigue. Over the last year, she complained of recurrent abdominal pain with suspicion of constipation. There was no weight loss and stools were normal. Her diet consisted mainly of whole milk. She was the first of two children of healthy, non‐consanguineous Dutch Moroccan parents. The family history was unremarkable, besides two nieces with IDA. Physical examination revealed pale skin and conjunctivae, a systolic murmur over the heart with a blood pressure of 115/65 mmHg, a pulse of 95 per minute, and a respiratory rate of 18 per minute. Otherwise, normal physical findings. The blood test showed severe microcytic anemia (Hb 5.1 g/dL, MCV 56 fL) and thrombocytopenia (platelets 18x10E9/L) with low ferritin (4 ng/mL). Additional blood work, including folic acid, vitamin B12, coeliac screening, and hemoglobin electrophoresis, was normal. Because two cell lines were down, a bone marrow investigation was performed, revealing the absence of iron staining and some dysplastic erythropoiesis and megakaryopoiesis. Oral iron medication was started (6 mg/kg/daily). The platelet count improved rapidly, while her hemoglobin recuperated more gradually.

#### 2.1.3 Patient 3

A 15‐year‐old male presented to the clinic because of paleness and fatigue. For a month he had suffered from watery diarrhea, 1‐2× daily, without fever, loss of appetite, or weight loss. No recent travels abroad. He dislikes eating vegetables but otherwise keeps a normal diet. Born at term by uncomplicated vaginal delivery after a normal pregnancy, he was the first of two children of healthy, non‐consanguineous Belgium‐Kenyan parents. The family history was unremarkable. Physical examination revealed a pale adolescent with a systolic heart murmur, mild splenomegaly, and otherwise normal findings (blood pressure 100/60 mmHg, pulse 88 per minute, respiratory rate 20 per minute). The blood test showed severe microcytic anemia (Hb 4.1 g/dL, MCV 55 fL) with low reticulocyte count and low ferritin (<1 ng/mL). Serology for cytomegalovirus, parvovirus, and Epstein‐Barr virus, G6PD level, and hemoglobin electrophoresis was normal. Genotyping of the α‐globin cluster revealed silent carrier status for alpha thalassemia (αα/α‐). Several days after starting oral iron treatment (6 mg/kg/daily), no increase of hemoglobin and reticulocyte count was seen, but thrombocytopenia (platelets 34×10E9/L) appeared. Because two cell lines were down, a bone marrow investigation was performed, revealing the complete absence of iron staining and increased erythropoiesis and megakaryopoiesis. Some dysplastic features (like nuclear segmentation) of the megakaryocytes were seen. The bone marrow puncture was conducted under general anesthesia; therefore, a red blood cell and platelet transfusion were given beforehand. The following day iron was administered intravenously to quickly improve his iron status. Upon discharge oral iron supplementation was restarted. The platelet count improved rapidly, while his hemoglobin recovered more gradually. The gastro‐intestinal evaluation did not reveal a cause of chronic blood loss.

#### 2.1.4 Patient 4

A 14‐year‐old female presented to the family physician for paleness. She had menorrhagia without complaints of abdominal pain or fatigue. She was the second of three children of healthy, non‐consanguineous Bulgarian‐Belgian parents. Medical history and family history were unremarkable, except that her sister and mother also suffered from menorrhagia. Physical examination revealed normal parameters and besides paleness no clinical abnormalities. The blood test showed severe microcytic anemia (Hb 6.7 g/dL, MCV 62 fL) and mild thrombocytopenia (platelets 87×10E9/L) with low ferritin (2 ng/mL). Additional blood work, including folic acid, vitamin B12, coagulation tests, and hemoglobin electrophoresis, was normal. Serology for cytomegalovirus, Epstein‐Barr virus, and parvovirus did not show any active disease. Oral iron medication was started (6 mg/kg/daily) and the platelet count improved quickly. On D27 after the start of oral iron treatment, the hemoglobin did not show any improvement, therefore, a red blood transfusion was given, and oral anti‐conceptive medication was added. Her hemoglobin recuperated to normal levels.

#### 2.1.5 Patient 5

A 17‐year‐old female was admitted to the hospital for balloon valvuloplasty for congenital aortic valve stenosis. Besides some fatigue, no clear complaints. Her diet was severely iron deficient, and she had menorrhagia. Until a few months ago, she used oral contraceptives for heavy periods. She was the first child of healthy, non‐consanguineous parents from Suriname. The family history was unremarkable. Physical examination revealed normal parameters (blood pressure 100/70 mmHg, pulse 95 per minute, respiratory rate 18 per minute), and except for paleness and a systolic heart murmur, no abnormalities. The blood test showed severe microcytic anemia (Hb 4.9 g/dL, MCV 62 fL) and thrombocytopenia (platelets 16×10E9/L) with low ferritin (7 ng/mL). Additional blood work, including coagulation tests, parvovirus serology, and hemoglobin electrophoresis, was normal. A bone marrow investigation was performed, revealing the absence of iron staining and an increased megakaryocyte count. She received red blood cell and platelet transfusions, and a one‐time gift of IV iron. Oral iron medication was started (5 mg/kg/daily). After the transfusions, her hemoglobin and platelet were raised to, respectively, 7.4 g/dL and 35x10E9/L. The platelet counts rapidly improved to normal value, while the hemoglobin started to increase more gradually.

## 3 RESULTS

The cases discussed, supplemented by cases from the literature, are summarized in Table 1. In 1964, Gross et al were the first who reviewed the platelets in IDA in children (not included in Table 1 due to the lack of clear case‐to‐case data). Their study population consisted of 60 previously untreated, iron‐deficient infants and children, of whom 6 children met the search criteria we set (hemoglobin 2.5‐4.5 g/dL, platelets 50‐100×10E9/L). Iron treatment increased hemoglobin and platelet count. A peak of the reticulocyte and the platelet count was seen around day 5 after parenteral iron treatment, while the reticulocyte count peaked around day 9 and the platelet count peaked around day 20 of oral iron treatment.^7^ The patients discussed and reported^6^, ^8^, ^9^, ^10^, ^11^, ^12^, ^13^, ^14^, ^15^, ^16^, ^17^, ^18^ in Table 1 all presented with severe IDA (median 4.5 g/dL, range 1.6‐6.9 g/dL) and thrombocytopenia (median 38×10E9/L, range 7‐100×10E9/L), both improving with iron treatment, which makes another cause such as Idiopathic Thrombocytopenic Purpura unlikely. There is no relationship between the severity of the anemia and the severity of the thrombocytopenia. Due to the severity of the anemia (67% Hb < 5 g/dL), in two‐third (67%) of the cases a red blood cell transfusion was given. No apparent hemodynamic instability was reported. Although none of the reported patients had signs of major bleeding, the thrombocytopenia could mostly (73%) be classified as severe (platelet count <50×10E9/L). In 13% of the cases, a platelet infusion was given. The peak of the platelet count (median 652×10E9/L, range 125‐1497×10E9/L, interpretation subject to change due to limited availability of data) was seen in the first month after the start of iron treatment. The exact moment of the peak is not known. In many cases (67%), a bone marrow examination was done, showing the absence of iron and a varying number of megakaryocytes. In the majority (61%, and in 4 of the 5 cases presented here), the bone marrow showed increased megakaryopoiesis. In cases 2 and 3, some dysplastic features were noted, but were not reassessed because after the iron therapy, the blood counts normalized and there was no longer a need for an additional bone marrow examination. We were unable to explain the dysplastic features. The myeloid: erythroid ratio is often not reported. In eight cases of children with IDA, the thrombocytopenia appeared after the supplementation of iron was started (see Table 1). This could happen after RBC transfusion, IV iron, and or PO iron was given, 3 till 21 days after the start of the therapy.

## 4 DISCUSSION

IDA is more often associated with mild to moderate thrombocytosis than with thrombocytopenia. The severity of thrombocytosis increases with the severity of anemia. There are conflicting opinions on whether or not cytokines are responsible for this (including interleukin‐6 and thrombopoietin).^19^, ^20^ Another possible contributing factor is the induction of erythropoietin in anemic patients.^21^ It stimulates the erythroid progenitor cells in the bone marrow to expand the erythroid lineage.^22^ It may additionally exhibit a synergistic effect on the platelet production due to some structural homology with thrombopoietin in the amino acid sequence or the expression of erythropoietin (EPO)‐receptors on the megakaryocyte progenitors.^23^, ^24^ The level of erythropoietin does, however, correlate poorly with the platelet count^20^ and is, therefore, unlikely to be the main contributing factor of thrombocytosis.^25^, ^26^, ^27^ This was confirmed in a study where IDA patients with chronic renal failure showed a resolution of their thrombocytosis when treated with IV iron therapy independent of their use of erythropoiesis‐stimulating agents.^24^ Eder et al examined 1273 anemic whole blood donors of whom 721 were iron depleted. This last group had significantly higher platelet counts, which decreased with iron therapy. While administered iron therapy in the control group did not affect platelet counts.^4^ These results indicate that there is an EPO‐independent link between iron deficiency and thrombocytosis. Xavier‐Ferrucio et al present experimental findings that support this hypothesis that low iron in the bone marrow, attenuates extracellular signal‐regulated kinase signaling, slow proliferation, and biases the commitment of megakaryocytic‐erythroid progenitors toward the megakaryocytic lineage in both human and mouse.^28^ This theory could explain the thrombocytosis observed in IDA.

Thrombocytopenia is much less common in IDA and is often associated with severe iron deficiency.^3^ It has been reported in children (see Table 1) and adults. In adults, three studies involving platelet count with IDA were published. Kadikoylu et al report 2 (2.3%) patients with thrombocytopenia in a population of 86 female patients with IDA at diagnosis.^29^ Sagar examined 172 women with IDA, in whom 4 (2.3%) had thrombocytopenia.^30^ Likewise, Kuku et al found thrombocytopenia in 13 (2.1%) of 615 adult patients with IDA.^5^ The mechanisms for the low platelet count are not well established, although the rapid improvement of the platelet count after iron administration, often even before the onset of reticulocytosis, suggests that iron may be necessary for platelet production.^31^ Severe iron deficiency may interfere with the folate and vitamin B12 utilization, as suggested by the megaloblastic changes in the bone marrow of some patients, despite normal levels of folic acid and vitamin B12.^32^ In severe iron deficiency, the bone marrow is exposed to high EPO levels, favoring the erythroid lineage at the expense of platelet production. However, the thrombocytopenia in IDA cannot be explained by a reduced number of megakaryocytes, as the bone marrow shows normal or elevated numbers of megakaryocytes,^13^, ^32^, ^33^ which was also demonstrated in our cases. We are unable to rule out this theory completely as the timing of the bone marrow examination in relation to the thrombocytopenia might be important, which could explain the normal and increased megakaryopoiesis in the cases (see Table 1). Iron could play a direct role in thrombopoiesis by the alteration in the activity of iron‐dependent enzymes.^12^, ^27^ Iron deficiency can increase megakaryopoietic differentiation and alter the platelet phenotype without changes of thrombopoietin^34^ but maybe important for platelet biogenesis and release.

Reports of thrombocytopenia after the start of iron replacement therapy, are extremely rare.^35^ In general, the platelet count returns to normal regardless of whether the iron therapy is interrupted or not. The timing of the appearance of thrombocytopenia after the start of iron therapy is variable, as well as normalization of the thrombocyte count and is unrelated to the method of iron administration (see also Table 1). The transient decrease in platelet counts following the start of iron therapy is possibly related to the phenomenon of stem cell steal. The sudden increased availability of iron may cause the pluripotent hematopoietic stem cell toward erythropoiesis, at the expense of the other hematopoietic cell lines. The effect of erythropoietin therapy on platelet counts is dependent on the adequacy of iron stores. When iron supply is lacking intense erythropoietin stimulation may cause thrombocytosis, but when the iron is amply present, erythropoiesis predominates and megakaryopoiesis may be transiently decreased.^12^, ^27^, ^36^, ^37^, ^38^ The ascertainment that mild iron deficiency is associated with mild thrombocytosis, while severe iron deficiency shows thrombocytopenia, suggests that iron may be required for thrombopoiesis.^31^

## 5 CONCLUSION

The combination of severe IDA and thrombocytopenia are reported in children. With this rarely occurring entity, the use of red blood cell transfusion and the performance of a bone marrow examination is common. Iron monotherapy shows improvement of the platelet count as well as the hemoglobin. Therefore, if clinically feasible and other causes are unlikely (no hepatosplenomegaly or normal white blood cell count and differentiation), empiric iron therapy is warranted, and bone marrow examination and red cell or platelet transfusion can be omitted.

## FUNDING

No funding was provided for this study.

## CONFLICT OF INTEREST

The authors have declared that there is no conflict of interest.

## AUTHOR CONTRIBUTIONS

Conceptualization: Machiel van den Akker

Formal Analysis: Machiel van den Akker

Writing – original draft: Machiel van den Akker, Laura Chielens, Lisa Lopes, Jaques van Heerden, and Mahmoud Zaqout

Writing – review and editing: Machiel van den Akker, Jutte van der Werf Ten Bosch

## AUTHOR APPROVAL AND DATA INTEGRITY

Authors grant Wiley a license to publish the article and identify itself as the original publisher. Authors also grant any third party the right to use the article freely as long as its integrity is maintained and its original authors, citation details and publisher are identified.

## TRANSPARENCY STATEMENT

This manuscript is an honest, accurate, and transparent account of the study being reported and no important aspects of the study have been omitted.
